# Biologer: an open platform for collecting biodiversity data

**DOI:** 10.3897/BDJ.8.e53014

**Published:** 2020-06-11

**Authors:** Miloš Popović, Nikola Vasić, Toni Koren, Ivona Burić, Nenad Živanović, Dejan Kulijer, Ana Golubović

**Affiliations:** 1 University of Niš, Faculty of Natural Sciences and Mathematics, Višegradska 33, 18000 Niš, Serbia University of Niš, Faculty of Natural Sciences and Mathematics Višegradska 33, 18000 Niš Serbia; 2 Singidunum University, Niš, Serbia Singidunum University Niš Serbia; 3 Association Hyla, Zagreb, Croatia Association Hyla Zagreb Croatia; 4 ZoozDev, Novi Sad, Serbia ZoozDev Novi Sad Serbia; 5 National Museum of Bosnia and Herzegovina, Sarajevo, Bosnia and Herzegovina National Museum of Bosnia and Herzegovina Sarajevo Bosnia and Herzegovina; 6 University of Belgrade, Faculty of Biology, Belgrade, Serbia University of Belgrade, Faculty of Biology Belgrade Serbia

**Keywords:** Eastern Europe, Balkan Peninsula, Serbia, Croatia, Bosnia and Herzegovina, Darwin Core, species observations, database

## Abstract

**Background:**

We have developed a new platform named "Biologer" intended for recording species observations in the field (but also from literature resources and collections). The platform is created as user-friendly, open source, multilingual software that is compatible with Darwin Core standard and accompanied by a simple Android application. It is made from the user’s perspective, allowing everyone to choose how they share the data. Project team members are delegated by involved organisations. The team is responsible for development of the platform, while local Biologer communities are engaged in data collection and verification.

**New information:**

Biologer has been online and available for use in Serbia since 2018 and was soon adopted in Croatia and Bosnia and Herzegovina. In total, we have assembled 536 users, who have collected 163,843 species observation records data from the field and digitalised 33,458 literature records. The number of active users and their records is growing daily. Out of the total number of gathered data, 89% has been made open access by the users, 10% is accessible on the scale of 10×10 km and only 1% is closed. In the future, we plan to provide a taxonomic data portal that could be used by local and national initiatives in Eastern Europe, aggregate all data into a single web location, create detailed data overview and enable fluent communication between users.

## Introduction

The study of biological diversity is one of the fields which vastly benefited from the development of modern computers and software tools (Bisby 2000, Dhillon and Sidhu 2013). Personal computers, digital cameras and smart phones enabled easier collection of field data, while powerful servers helped to systematise and analyse these large datasets. Over the years, Global Biodiversity Information Facility (GBIF) became one of the best known software platforms that collects open biodiversity data from around the globe, amassing more than a thousand million records so far (Gilman et al. 2009), but there are many other software platforms for this purpose (Costello et al. 2014). In the open science area (i.e. Walsh 2016), there are several open software solutions to collect data (e.g. Indicia, Specify, iNaturalist) and publish them in the open access manner (Chavan and Penev 2011, Smith et al. 2011, Vattakaven et al. 2016). However, not all the regions of the planet are equally covered by collected biodiversity data, with eastern and south-eastern Europe being under-represented globally (Wetzel et al. 2018, GBIF map of global coverage). Despite all the progress in the developed world, it seems that most of the species observations from this part of Europe remained within hardcopies of field notebooks and will likely never be analysed, published or used.

In Serbia, the first tools for collecting observation data were created for insects (Alciphron software, Đurić 2005) and aquatic biodiversity (Simić et al. 2006). These were followed by an initiative to create a unique database of biological diversity of Serbia – the BioRaS platform (Mesaroš et al. 2014) and, later on, by an online version of Alciphron (HabiProt 2014). In Croatia, the long-term working database was established for collecting plant species observations (Fertalj et al. 2000), while several new initiatives emerged to collect data on marine life (Blue World 2015, Gomerčić and Đuras 2017) and birds (BIOM 2017). All of these software solutions were proprietary and their development usually slowed down after the funding ended, while the work on the most promising, BioRaS platform, was completely abandoned. More importantly, in all these early initiatives, data are usually owned by a single organisation or a few people and it was hard to legally obtain verifiable data for scientific purposes. Beside these regional datasets, some individuals are involved in global initiatives (most active being iNaturalist, eBird, NaturaList, Observation.org). These platforms sometimes provide open data to the scientific community, but over the years, we have faced problems with incomplete taxonomy, lack of local expertise, inadequate software localisation or lack of some important data (i.e. the level of precision of the coordinates). These problems could easily be solved by managing biodiversity data through local initiatives and serving them to the global biodiversity information platforms using common standards.

Having experienced common problems with existing database solutions and exploring the ways to deal with them (Costello et al. 2014), we wanted to develop a sustainable, easy-to-use and open source platform, giving users the ability to choose how to share collected data and photos. A network of people and organisations involved in biodiversity and nature conservation has already been established in Eastern Europe through various projects and activities and can be used to promote Biologer.

## Project description

### Title

Biologer

### Design description

**Figure 1.**
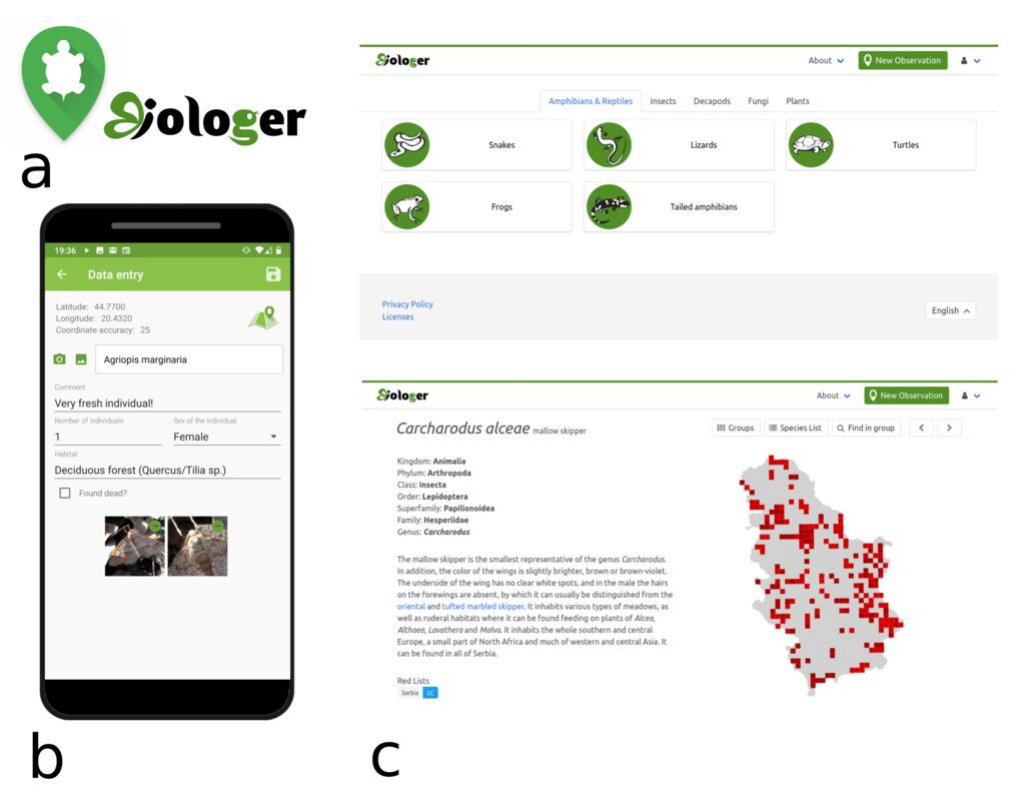
Overview of the Biologer interfaces showing: a) icon and project logo design, b) mobile application for Android devices with detailed data entry form and c) web interface displaying available groups (top) and a single butterfly species view (bottom).

**Figure 2.**
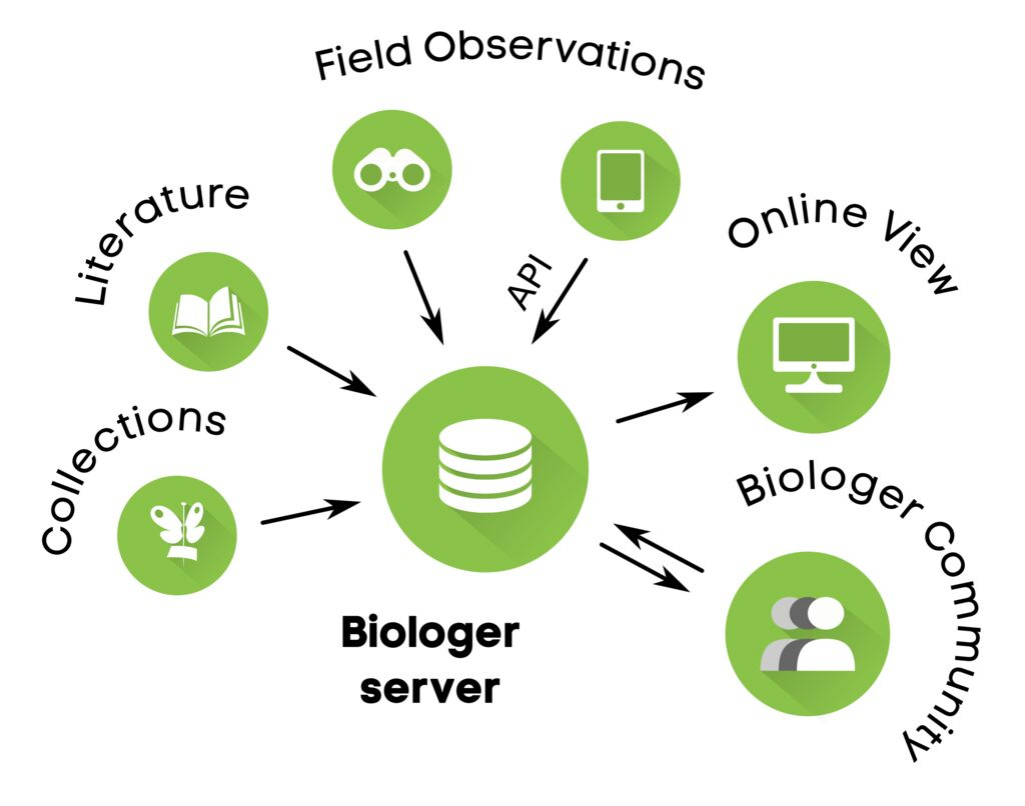
The concept of Biologer platform organisation. Server is in charge of gathering data from the web interface (field observations, scientific literature or collections) or through API endpoint, accessed by a smart phone device running Android (field observations only). The collected data are moderated by Biologer community (users, editors and administrators) and displayed online as simple biodiversity encyclopaedia.

The Biologer title is derived from our local languages by joining words "biologija" (biology) and "loger" (logger). It follows a simple and user-friendly design, with logo and icons clearly associating with biological species and geographic information system (Fig. 1). The main component of Biologer platform is an online software that handles users, taxa and species occurrence records, while providing data view, data import/export and API endpoint for communicating with other software (Fig. 2). Sharing and licensing of data are completely in the hands of Biologer users. Editors and administrators are able to access all data from the groups they moderate, but data usage is clearly defined by licences, privacy policy and local community rules displayed online. One of the main project goals is to promote open software and open data and use this data in nature conservation programmes.

### Funding

Rather than being owned by an individual or organisation, we chose to create a Project team responsible for further development of Biologer. A growing community of scientific and civil society organisations is participating in the development by nominating people for the Project team and providing fundings and other resources. Initial development of Biologer was supported by the Rufford Small Grants (projects No: 20507-B and 24652-B). Additional support to implement a database solution in Croatia was received from the MAVA foundation. The work of MP and AG is partially supported through the Ministry of Education, Science and Technological Development of the Republic of Serbia (projects No: 173025, 173043, 451-03-68/2020-14/200124 and 451-03-68/2020-14/200178). A significant part of the work is done on a voluntary basis through engagement of the Biologer community.

## Web location (URIs)

Homepage: https://biologer.org

Download page: https://github.com/Biologer

## Technical specification

Platform: Laravel framework (web) and Android Studio (Android)

Programming language: PHP (web) and Java (Android)

Operational system: Designed for GNU/Linux and Android, but should also work on other operating systems.

Interface language: English, Serbian, Croatian, Bosnian. Hungarian is available only on Android.

## Usage rights

### Use license

Creative Commons Public Domain Waiver (CC-Zero)

## Implementation

### Implements specification

The online software is the main part of Biologer (Fig. 2) handling 1) user registration and privileges (being users, editors and administrators), 2) taxonomic database (list of taxa, their localised names, descriptions, threat and conservation status), 3) species observations (data entry, verification and publication) and 4) communication with other software through a web service. An Android application was also developed for use during the fieldwork (Fig. 1b). It enables easy data entry by automatically filling user data, location with coordinates and coordinate precision, date and time of each observation. Besides field observations, the software allows editors to enter species observations digitalised from available scientific literature, while the entry for collection data is in the final phase of development. Easy transition from other software solutions is possible, since Biologer offers the option to import structured data from CSV tables.

An important step in publishing biodiversity data is the review process (Costello et al. 2013), which involves taxonomic experts (editors) from the Biologer community. They are responsible for checking each observation, approving the identification of taxa (species complex, species or subspecies) or marking the finding as unidentifiable. Unidentifiable records could be supplemented later on with detailed evidence for species observations (i.e. images of certain body parts) or reviewed and identified by more experienced editors. To track these activities, the history of changes is logged along with each species observation.

Data access is principally controlled by the users’ settings defining separate licences for images and observations, but we strongly suggest choosing open data for its greater applicability in both science and nature conservation (Groom et al. 2016). Access can also be restricted by the editors for particular taxa, if the survival of endangered local populations could be threatened by showing detailed occurrences on the maps. The species observations for these restricted taxa is shown as 10×10 km square, without precise coordinates. All species observations with open access can be viewed from the Contributor Area and saved as Comma Separated Value (CSV) file by registered users. In order to enhance cooperation with existing biodiversity portals, data can be exported using standardised Darwin Core terms (Wieczorek et al. 2012). Data from Biologer was successfully used in several projects, ranging from collecting species observations of dragonflies, reptiles and butterflies, reporting on butterfly species for Emerald network, to providing data for the desktop analysis in designating Natura 2000 in Serbia (EuropeAid/139336/DH/SER/RS). We have already used Biologer as the prime source of data for analysing distribution of Hermann’s tortoise (Golubović et al. 2019) and brown frogs (Urošević et al. 2018) and for updating the Red List of butterflies of Serbia (published in Maes et al. 2019).

Finally, Biologer offers a simple data browser from its main page (Fig. 1c). Each species overview is designed as an online encyclopaedia providing scientific and vernacular name, short descriptions, threat and legal status on national and international level, phenological graph, map of records within the country and a gallery of photos. Species descriptions are published with a Creative Commons licence to promote sharing of intellectual knowledge and are being updated by the editors.

**Future perspectives**

With the growing support from several local communities, one of our prime goals is to make the taxonomic database a separate component of the platform. The taxonomic database will provide a list of synonyms, country specific checklists, conservation and legal status for each country involved, local species names and descriptions, as well as an endpoint for communication with other components of Biologer. After completion of this task, we will be able to provide a single multilingual encyclopaedia on regional species diversity along with a content aggregation of open access data from all officially-involved Biologer communities on a single data server. Aggregated observations will be free to download or redistribute to global biodiversity databases according to the licences chosen by the users. We plan to enhance user experience by showing usage statistics, providing more detailed data overview and facilitating direct communication amongst users. Biologer could also support adding different types of data, such as species counts along transects or within delineated areas (polygons).

### Audience

Biologer has been collecting observation data from Serbia since 2018 at biologer.org. Croatia joined our initiative registering at biologer.hr in 2019, while in 2020 Bosnia and Herzegovina started their data portal at biologer.ba. Currently, each country maintains its own server for data collecting and has its own community of users, while the Biologer application for Android devices allows choosing the prefered community. At the time of writing this manuscript, we have gathered 536 users (64% from Serbia, 25% from Croatia and 11% from Bosnia and Herzegovina) and collected 163,843 records from the field (Serbia 71%, Croatia 27%, Bosnia and Herzegovina 2%). Additionally, the Serbian data portal imported 33,458 digitalised literature records. It is interesting to note that users predominantly choose open data licences for their observation records. Most of the records (89%) are delivered under open access licences, 10% of data is accessible on the scale of 10×10 km, while only 1% of data is closed. Being issued under the MIT licence, Biologer software is available to the public and any organisation or individual could run it on a dedicated server and modify it for their own needs. A good example for this is the "Biologer Otis" application adapted for monitoring the great bustard (*Otis tarda*) population in Serbia. Our Project team will work on acquiring more developers and volunteers and establishing other local Biologer communities within the region.

## References

[B5635095] BIOM fauna.hr. https://www.fauna.hr.

[B5669947] Bisby F. A. (2000). The quiet revolution: Biodiversity informatics and the Internet. Science.

[B5635079] World Blue Marine partnership. https://www.partnerstvozamore.org/en/.

[B5633173] Chavan Vishwas, Penev Lyubomir (2011). The data paper: a mechanism to incentivize data publishing in biodiversity science. BMC Bioinformatics.

[B5633721] Costello Mark J., Michener William K., Gahegan Mark, Zhang Zhi-Qiang, Bourne Philip E. (2013). Biodiversity data should be published, cited, and peer reviewed. Trends in Ecology & Evolution.

[B5633431] Costello Mark J., Appeltans Ward, Bailly Nicolas, Berendsohn Walter G., de Jong Yde, Edwards Martin, Froese Rainer, Huettmann Falk, Los Wouter, Mees Jan, Segers Hendrik, Bisby Frank A. (2014). Strategies for the sustainability of online open-access biodiversity databases. Biological Conservation.

[B5633497] Dhillon Sarinder K., Sidhu Amandeep S. (2013). Data intensive computing for biodiversity.

[B5635205] Đurić Milan (2005). Alciphron software for Windows.

[B5633393] Fertalj Krešimir, Nikolić Toni, Helman Tomo, Mornar Vedran, Kalpić Damir, Mastorakis Nikos E. (2000). Flora Croatica Database Application. Mathematics and Computers in Modern Science.

[B5633143] Gilman Eric, King Nicholas, Peterson A. Townsend, Chavan Vishwas, Hahn Andrea, Maurer Lisa (2009). Building the biodiversity data commons: The Global Biodiversity Information Facility. ICT for Biodiversity Conservation and Agriculture.

[B5638829] Golubović Ana, Tomović Ljiljana, Nikolić Marko, Nikolić Sonja, Andjelković Marko, Arsovski Dragan, Iković Vuk, Gvozdenović Sladjana, Popović Miloš (2019). Distribution of Hermann’s tortoise across Serbia with implications for conservation. Archives of Biological Sciences.

[B5635113] Gomerčić Tomislav, Đuras Martina CROdolphin. http://crodolphin.vef.hr.

[B5666566] Groom Quentin, Weatherdon Lauren, Geijzendorffer Ilse R. (2016). Is citizen science an open science in the case of biodiversity observations?. Journal of Applied Ecology.

[B5618740] HabiProt Alciphron - database on insects of Serbia. http://www.alciphron.habiprot.org.rs.

[B5647068] Maes Dirk, Verovnik Rudi, Wiemers Martin, Brosens Dimitri, Beshkov Stoyan, Bonelli Simona, Buszko Jaroslaw, Cantú-Salazar Lisette, Cassar Louis-Francis, Collins Sue, Dincă Vlad, Djuric Milan, Dušej Goran, Elven Hallvard, Franeta Filip, Garcia-Pereira Patricia, Geryak Yurii, Goffart Philippe, Gór Ádám, Hiermann Ulrich, Höttinger Helmut, Huemer Peter, Jakšić Predrag, John Eddie, Kalivoda Henrik, Kati Vassiliki, Kirkland Paul, Komac Benjamin, Kőrösi Ádám, Kulak Anatolij, Kuussaari Mikko, L’Hoste Lionel, Lelo Suvad, Mestdagh Xavier, Micevski Nikola, Mihoci Iva, Mihut Sergiu, Monasterio-León Yeray, Morgun Dmitry V., Munguira Miguel L., Murray Tomás, Nielsen Per Stadel, Ólafsson Erling, Õunap Erki, Pamperis Lazaros N., Pavlíčko Alois, Pettersson Lars B., Popov Serhiy, Popović Miloš, Pöyry Juha, Prentice Mike, Reyserhove Lien, Ryrholm Nils, Šašić Martina, Savenkov Nikolay, Settele Josef, Sielezniew Marcin, Sinev Sergey, Stefanescu Constanti, Švitra Giedrius, Tammaru Toomas, Tiitsaar Anu, Tzirkalli Elli, Tzortzakaki Olga, van Swaay Chris A. M., Viborg Arne Lykke, Wynhoff Irma, Zografou Konstantina, Warren Martin S. (2019). Integrating national Red Lists for prioritising conservation actions for European butterflies. Journal of Insect Conservation.

[B5618792] Mesaroš Gabor, Popović Miloš, Janev Srđan BioRaS: Biological diversity of Serbia. http://bioras.petnica.rs.

[B5635056] Simić V., Simić A., Petrović A., Paunović M., Šorić M., Dimitrijević V. Biodiversity of aquatic ecosystems of Serbia, *ex situ* conservation (BAES *ex situ*). http://baes.pmf.kg.ac.rs.

[B5633449] Smith Vincent, Rycroft Simon, Brake Irina, Scott Ben, Baker Ed, Livermore Laurence, Blagoderov Vladimir, Roberts David (2011). Scratchpads 2.0: a Virtual Research Environment supporting scholarly collaboration, communication and data publication in biodiversity science. ZooKeys.

[B5647043] Urošević Aleksandar, Tomović Ljiljana, Krizmanić Imre, Anđelković Marko, Golubović Ana, Maričić Marko, Ajtić Rastko, Ćorović Jelena, Čubrić Tijana, Tomašević-Kolarov Nataša, Cvijanović Milena, Vukov Tanja, Jovanović Bogdan, Vučić Tijana, Ajduković Maja, Tot Ivan, Nadaždin Bojana, Labus Nenad, Džukić Georg (2018). Distribution and diversity of brown frogs (*Rana* spp., Anura, Amphibia) in Serbia. Bulletin of the Natural History Museum.

[B5635179] Vattakaven Thomas, George Rohit, Balasubramanian Dhandapani, Réjou-Méchain Maxime, Muthusankar Gowrappan, Ramesh Brahmasamudra, Prabhakar R (2016). India Biodiversity Portal: An integrated, interactive and participatory biodiversity informatics platform. Biodiversity Data Journal.

[B5633202] Walsh K. (2016). Open innovation, open science, open to the world – a vision for Europe.

[B5669927] Wetzel Florian T., Bingham Heather C., Groom Quentin, Haase Peter, Kõljalg Urmas, Kuhlmann Michael, Martin Corinne S., Penev Lyubomir, Robertson Tim, Saarenmaa Hannu, Schmeller Dirk S., Stoll Stefan, Tonkin Jonathan D., Häuser Christoph L. (2018). Unlocking biodiversity data: Prioritization and filling the gaps in biodiversity observation data in Europe. Biological Conservation.

[B5635413] Wieczorek John, Bloom David, Guralnick Robert, Blum Stan, Döring Markus, Giovanni Renato, Robertson Tim, Vieglais David (2012). Darwin Core: An Evolving Community-Developed Biodiversity Data Standard. PLOS One.

